# Supplementary material for: The structural basis for RNA slicing by human Argonaute2
Source: Cell Rep. Author manuscript; Available in PMC 2025 Mar 10. (PMC11893014; doi:10.1016/j.celrep.2024.115166)
Supplement: 1 [file NIHMS2052760-supplement-1.pdf]

Cell Reports, Volume *44*

Supplemental information

The structural basis for RNA slicing by human Argonaute2

Abdallah A. Mohamed, Peter Y. Wang, David P. Bartel, and Seychelle M. Vos

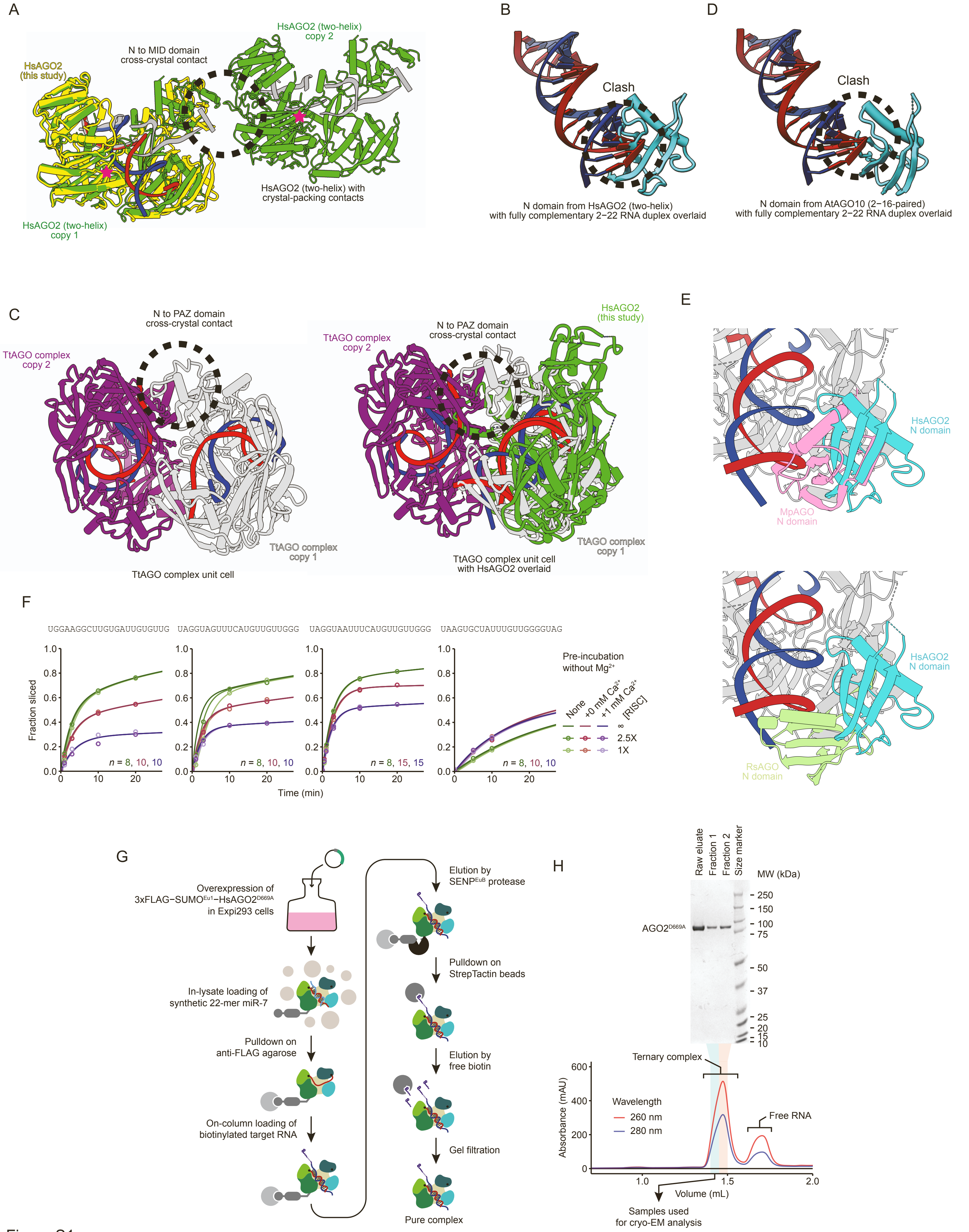

A
HsAGO2 (two-helix) copy 2
N to MID domain cross-crystal contact
HsAGO2 (this study)
HsAGO2 (two-helix) with crystal-packing contacts
HsAGO2 (two-helix) copy 1
B
Clash
N domain from HsAGO2 (two-helix) with fully complementary 2−22 RNA duplex overlaid
D
Clash
N domain from AtAGO10 (2−16-paired) with fully complementary 2−22 RNA duplex overlaid
C
N to PAZ domain cross-crystal contact
TtAGO complex copy 2
TtAGO complex copy 1
TtAGO complex unit cell
N to PAZ domain cross-crystal contact
TtAGO complex copy 2
HsAGO2 (this study)
TtAGO complex copy 1
TtAGO complex unit cell with HsAGO2 overlaid
E
HsAGO2 N domain
MpAGO N domain
HsAGO2 N domain
RsAGO N domain
F
UGGAAGGCUUGUGAUUGUGUUG
UAGGUAGUUUCAUGUUGUUGGG
UAGGUAAUUUCAUGUUGUUGGG
UAAGUGCUAUUUGUUGGGGUAG
Fraction sliced
Time (min)
n = 8, 10, 10
n = 8, 10, 10
n = 8, 15, 15
n = 8, 10, 10
Pre-incubation without Mg2+
None
+0 mM Ca2+
+1 mM Ca2+
[RISC]
∞
2.5X
1X
G
Overexpression of 3xFLAG−SUMOEu1−HsAGO2D669A in Expi293 cells
In-lysate loading of synthetic 22-mer miR-7
Pulldown on anti-FLAG agarose
On-column loading of biotinylated target RNA
Elution by SENPEuB protease
Pulldown on StrepTactin beads
Elution by free biotin
Gel filtration
Pure complex
H
Raw eluate
Fraction 1
Fraction 2
Size marker
MW (kDa)
250
150
100
75
50
37
25
20
15
10
AGO2D669A
Ternary complex
Free RNA
Absorbance (mAU)
Wavelength
260 nm
280 nm
Volume (mL)
Samples used for cryo-EM analysis

Figure S1

Figure S1. Structural comparisons and purification of the HsAGO2−miR-7−target ternary complex, related to Figures 1 and 2.

(A) Crystal-packing contacts for HsAGO2 RISC in the two-helix conformation (PDB: 6N4O [S1]). N and MID domains form packing contacts between two copies of HsAGO2 RISC in the two-helix conformation (colored green). The fully paired HsAGO2 structure (yellow) is overlaid, showing that it is in a conformation that would disrupt these packing interactions. The active sites are indicated as magenta stars.

(B) Clash observed when the N domain from the HsAGO2 two-helix structure (PDB: 6N4O [S1]) is overlaid with a 21-bp RNA duplex model generated in ChimeraX bound within the central channel.

(C) Crystal contacts for TtAGO complex with a complementary target. On the left is the asymmetric unit of TtAGO, which contains two copies of TtAGO (colored gray and purple) (PDB: 4NCB [S2]), which form contacts between the N and PAZ domains. On the right, the fully paired HsAGO2 structure (green) is overlaid, showing that it would not accommodate the packing contacts.

(D) Clash observed when the N domain from the AtAGO10 2−16-paired structure (PDB: 7SWF [S3]) is overlaid with a 21-bp RNA duplex model generated in ChimeraX bound within the central channel.

(E) At the top is the N domain of MpAGO bound to an RNA−DNA hybrid fully paired to position 20 (PDB: 5UXO [S4]) (pink); the fully paired HsAGO2 structure is overlaid with the N domain colored in cyan. At the bottom is the same but for the N domain of RsAGO bound to an RNA−DNA hybrid fully paired to position 18 (PDB: 5AWH [S5]) (light green).

(F) Fraction of perfectly complementary target RNA sliced over time after preincubation in buffer without Mg^{2+}, either with or without Ca^{2+}, across different RISC concentrations, with four different guide RNAs loaded (indicated above). Solid lines represent best-fit lines from fitting to the ordinary differential equation system, alongside an extrapolated reaction curve at infinite RISC concentration, which represents a reaction rate determined by only k_{slice} and not k_{on}. Number of data points for each set is indicated as n. Time points beyond the limits of the x axes are not shown.

(G) Purification scheme for the HsAGO2^{D669A}−miR-7−target ternary complex.

(H) Size-exclusion chromatography of the ternary complex on a Superdex 200 3.1/200 column. Regular 50-µL fractions were collected by the chromatography system, which captured the ternary-complex peak in two fractions. These fractions were analyzed on an SDS-polyacrylamide gel, visualizing protein with Imperial (Coomassie R-250) staining, and used to make grids for cryo-EM analysis. Irrelevant lanes on the left of the gel are removed.

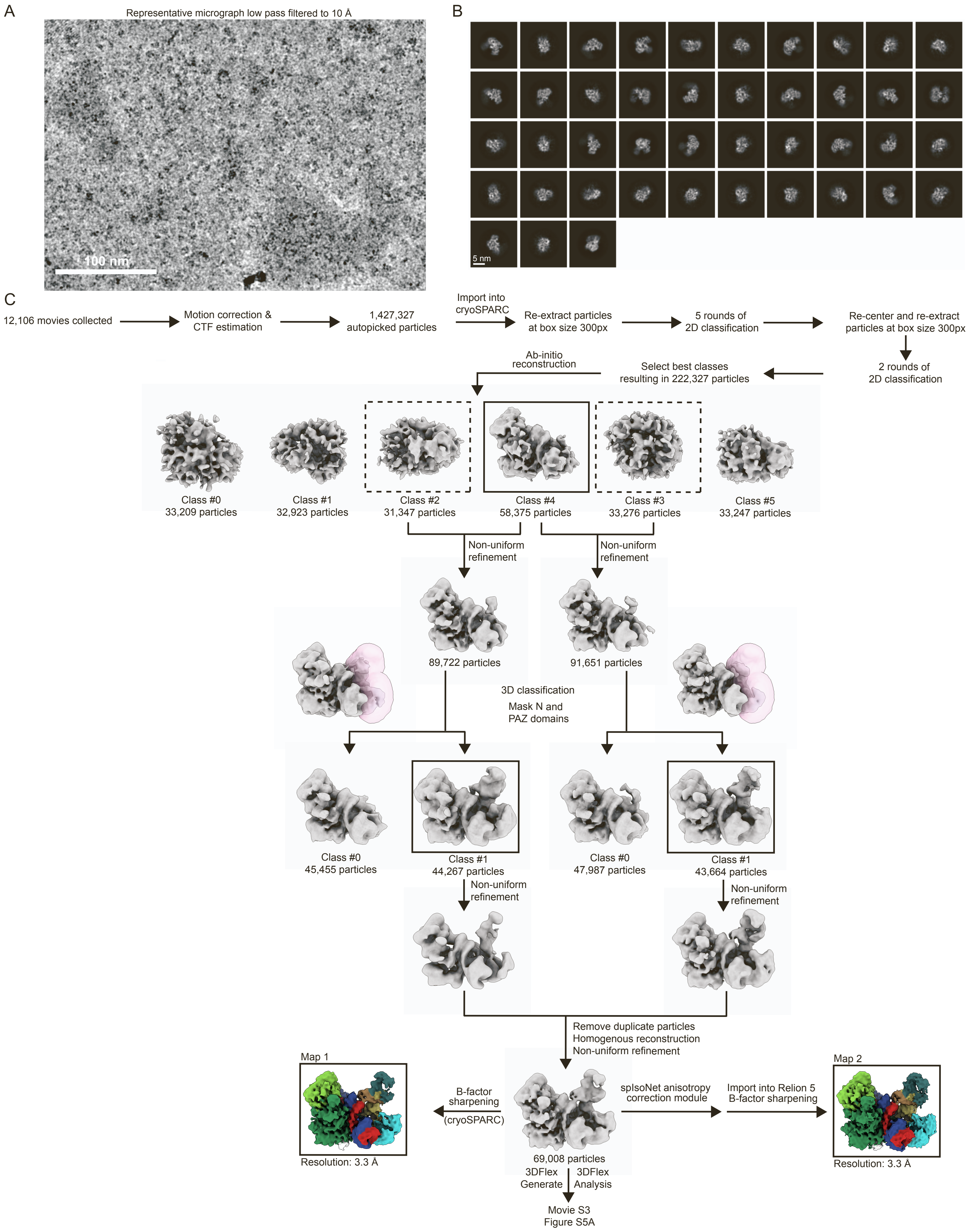
A
Representative micrograph low pass filtered to 10 Å
100 nm
B
5 nm
C
12,106 movies collected
Motion correction & CTF estimation
1,427,327 autopicked particles
Import into cryoSPARC
Re-extract particles at box size 300px
5 rounds of 2D classification
Re-center and re-extract particles at box size 300px
2 rounds of 2D classification
Select best classes resulting in 222,327 particles
Ab-initio reconstruction
Class #0 33,209 particles
Class #1 32,923 particles
Class #2 31,347 particles
Class #4 58,375 particles
Class #3 33,276 particles
Class #5 33,247 particles
Non-uniform refinement
Non-uniform refinement
89,722 particles
91,651 particles
3D classification
Mask N and PAZ domains
Class #0 45,455 particles
Class #1 44,267 particles
Class #0 47,987 particles
Class #1 43,664 particles
Non-uniform refinement
Non-uniform refinement
Remove duplicate particles
Homogenous reconstruction
Non-uniform refinement
Map 1
Resolution: 3.3 Å
B-factor sharpening (cryoSPARC)
69,008 particles
spIsoNet anisotropy correction module
Import into Relion 5 B-factor sharpening
Map 2
Resolution: 3.3 Å
3DFlex Generate
3DFlex Analysis
Movie S3
Figure S5A

Figure S2

Figure S2. Cryo-EM data collection and processing, related to Figures 1 and 3.
(A) Representative micrograph low-pass filtered to 10 Å. This micrograph is representative of 12,106 micrographs. Scale bar is shown at the bottom left.
(B) Representative 2D classes. Scale bar is shown.
(C) Classification tree for determining the structure of HsAGO2 in a slicing-competent conformation.

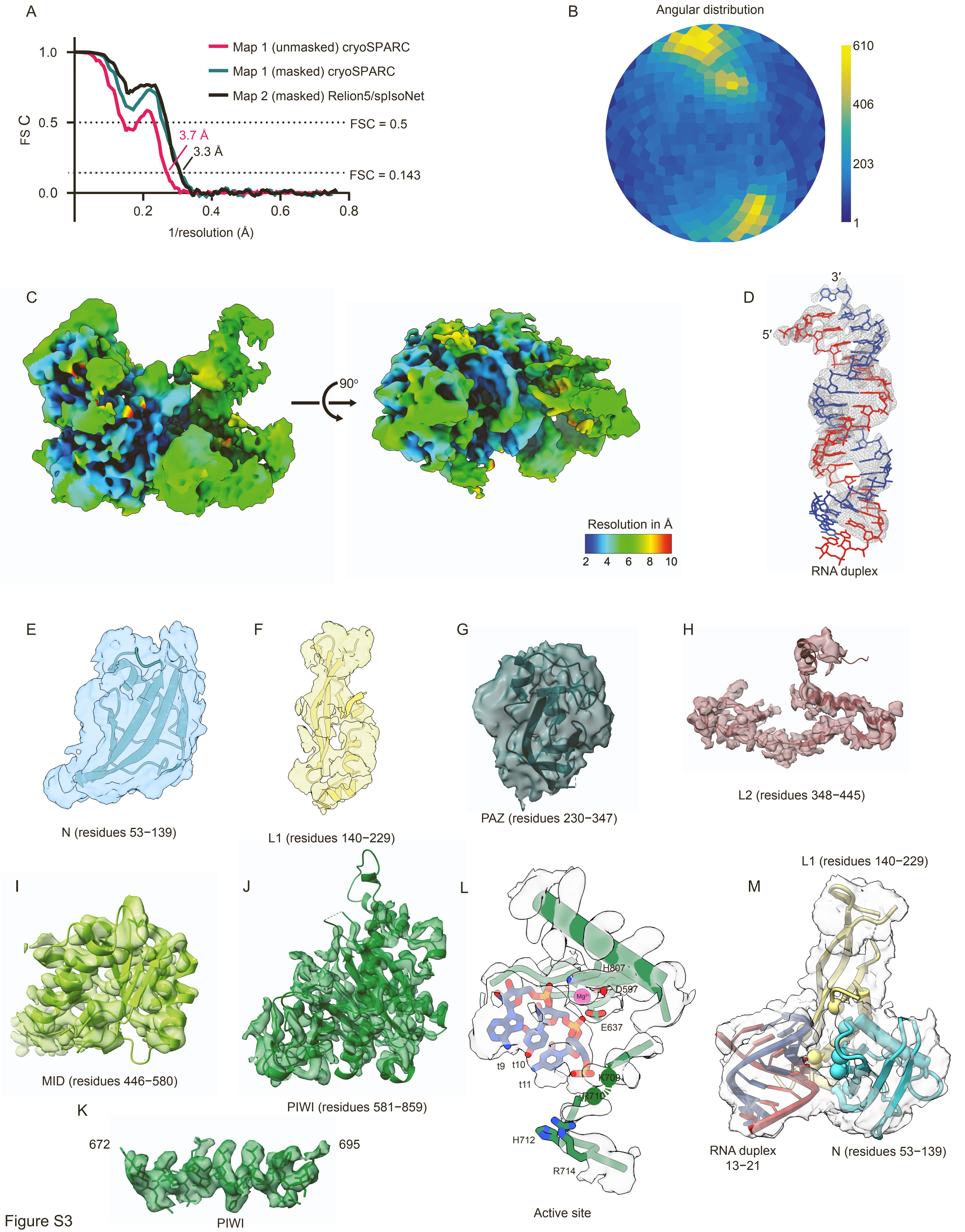
A
1.0
0.5
0.0
FSC
Map 1 (unmasked) cryoSPARC
Map 1 (masked) cryoSPARC
Map 2 (masked) Relion5/spIsoNet
FSC = 0.5
FSC = 0.143
3.7 Å
3.3 Å
0.2
0.4
0.6
0.8
1/resolution (Å)
B
Angular distribution
610
406
203
1
C
90°
Resolution in Å
2
4
6
8
10
D
3′
5′
RNA duplex
E
N (residues 53−139)
F
L1 (residues 140−229)
G
PAZ (residues 230−347)
H
L2 (residues 348−445)
I
MID (residues 446−580)
J
PIWI (residues 581−859)
K
672
695
PIWI
L
H807
D597
Mg²⁺
E637
t9
t10
t11
K709
R710
H712
R714
Active site
M
L1 (residues 140−229)
RNA duplex
13−21
N (residues 53−139)

Figure S3

Figure S3. Quality and resolution of cryo-EM data, related to Figures 1, 2, 4, and 5.
(A) Estimate of average resolution. Dotted lines indicate Fourier shell correlation (FSC) of 0.5 and 0.143. Solid lines indicate FSC between half-maps of the reconstruction.
(B) Angular distribution plot for particles used to reconstruct Map 1. Shading from blue to yellow indicates the number of particles at a given orientation.
(C) Reconstruction of the fully paired HsAGO2 complex, colored by local resolution (Map 1).
(D) Model of the RNA duplex in density from Map 2.
(E) Model of the N domain in density from Map 2.
(F) Model of the L1 domain in density from Map 2.
(G) Model of the PAZ domain in density from Map 2.
(H) Model of the L2 domain in density from Map 2.
(I) Model of the MID domain in density from Map 2.
(J) Model of the PIWI domain in density from Map 2.
(K) Model of PIWI-domain residues 672−695 in density from Map 2.
(L) Model of active site in density from Map 2.
(M) Model of the L1 and N domains, with positions 13−22 of the RNA duplex, in density from Map 2.

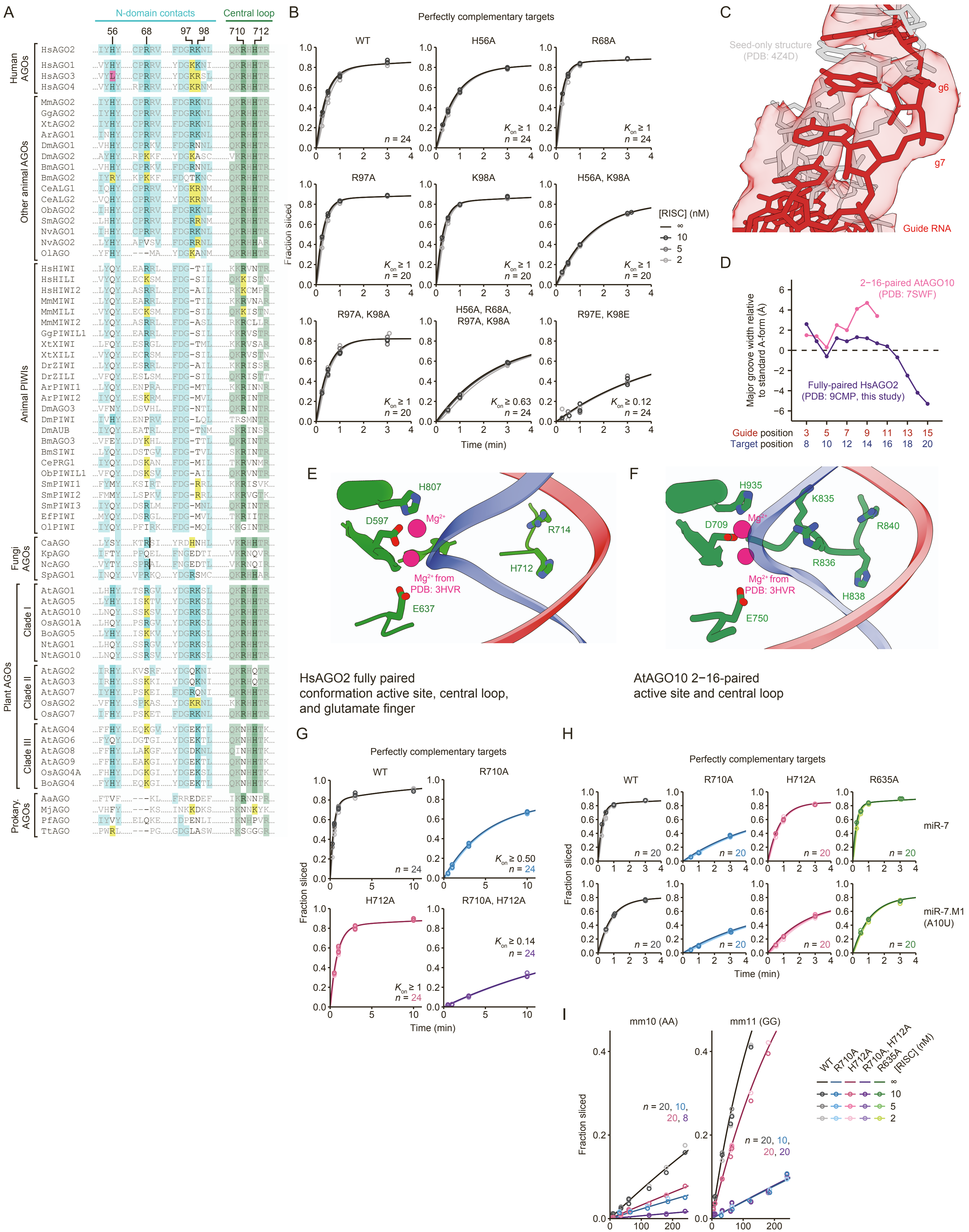
A
N-domain contacts
Central loop
Human AGOs
Other animal AGOs
Animal PIWIs
Fungi AGOs
Plant AGOs
Clade I
Clade II
Clade III
Prokary. AGOs
B
Perfectly complementary targets
WT
H56A
R68A
R97A
K98A
H56A, K98A
R97A, K98A
H56A, R68A, R97A, K98A
R97E, K98E
Fraction sliced
Time (min)
[RISC] (nM)
C
Seed-only structure (PDB: 4Z4D)
g6
g7
Guide RNA
D
2–16-paired AtAGO10 (PDB: 7SWF)
Fully-paired HsAGO2 (PDB: 9CMP, this study)
Major groove width relative to standard A-form (Å)
Guide position
Target position
E
H807
D597
Mg2+
R714
H712
Mg2+ from PDB: 3HVR
E637
F
H935
K835
D709
R840
R836
H838
E750
HsAGO2 fully paired conformation active site, central loop, and glutamate finger
AtAGO10 2–16-paired active site and central loop
G
Perfectly complementary targets
WT
R710A
H712A
R710A, H712A
Fraction sliced
Time (min)
H
Perfectly complementary targets
WT
R710A
H712A
R635A
miR-7
miR-7.M1 (A10U)
Fraction sliced
Time (min)
I
mm10 (AA)
mm11 (GG)
Fraction sliced
Time (min)
WT
R710A
H712A
R710A, H712A
R635A
[RISC] (nM)

Figure S4

Figure S4. Residues in N and PIWI domains regulate HsAGO2 slicing activity, related to Figures 2, 3, and 5.

(A) Representative multiple-sequence alignment of select AGO and PIWI homologs at the proposed N-domain and central-loop contacts. Residues identical to HsAGO2 are shaded in teal or green. Residues that changed from HsAGO2 but remained basic are shaded in yellow. An H56L substitution in HsAGO3 is shaded in pink. Insertions near R68 in two fungal homologs (one and five residues respectively) are indicated with black bars and otherwise omitted for simplicity. Species codes shown are as follows. For animals: Hs, *Homo sapiens* (human); Mm, *Mus musculus* (mouse); Gg, *Gallus gallus* (chicken); Xt, *Xenopus tropicalis* (frog); Dr, *Danio rerio* (zebrafish); Ar, *Asterias rubens* (star fish); Dm, *Drosophila melanogaster* (fruit fly); Bm, *Bombyx mori* (silkmoth); Ce, *Caenorhabditis elegans* (nematode); Ob, *Octopus bimaculoides* (octopus); Sm, *Schmidtea mediterranea* (flatworm); Nv, *Nematostella vectensis* (starlet sea anemone); Ef, *Ephydatia fluviatilis* (river sponge); Ol, *Oscarella lobularis* (sea sponge). For fungi: Ca, *Candida albicans*; Kp, *Kuyveromyces polysporus*; Nc, *Naumovozyma castellii*; Sp, *Schizosaccharomyces pombe* (fission yeast). For plants: At, *Arabidopsis thaliana*; Os, *Oryza sativa* (rice); Bo, *Brassica oleracea* (wild cabbage); Nt, *Nicotiana tabacum* (tobacco). For prokaryotes: Aa, *Aquifex aeolicus*; Mj, *Methanocaldococcus jannaschii*; Pf, *Pyrococcus furiosus*; Tt, *Thermus thermophilus*.

(B) Fraction of target RNA sliced over time by either wildtype (WT) or N-domain mutants of HsAGO2−miR-7, across different RISC concentrations (gray gradient). Solid lines represent best-fit lines from fitting to the ordinary differential equation system. The extrapolated reaction curve at infinite RISC concentration, which represents a reaction rate determined by only k_{slice} and not k_{on}, is plotted in black. Number of data points for each set is indicated as *n*. The lower limit of k_{on} is indicated as its ratio to the k_{on} observed for wildtype HsAGO2 (K_{on}), determined to a maximum value of 1. Time points beyond the limits of the *x* axes are not shown.

(C) Model and map of the seed region of the guide in the fully paired conformation, colored in red, showing the backbone kink between positions 6 and 7. The model of HsAGO2 paired only at the seed region (PDB: 4Z4D [S6]) is overlaid in gray.

(D) Analysis of major-groove width deviation from A-form of the RNA duplex in HsAGO2 in the fully paired conformation and the AtAGO10 2−16-paired conformation (PDB: 7SWF [S3]). Approximate positions of nearest backbone phosphates across the groove are indicated for guide and target strands respectively.

(E) Active site and central loop of HsAGO2 in the fully paired conformation. The position of the second Mg^{2+} ion, modeled based on a slicing-competent structure of TtAGO (PDB: 3HVR [S7]), is shown. Colors are as in **Figure 4D**.

(F) Active site and central loop of AtAGO10 in the 2−16-paired conformation (PDB: 7SWF [S3]). Otherwise, as in **E**.

(G) Fraction of target RNA sliced over time by either wildtype (WT) or central-loop mutants, across different RISC concentrations. Colors are as in **Figure 5A**; otherwise, as in **B**. Values with WT HsAGO2 are replotted from **B** for reference.

(H) Fraction of target RNA sliced over time by either wildtype (WT) or mutants of HsAGO2, loaded with either wildtype miR-7 or a mutant (miR-7.M1) that has a substitution at position 10, across different RISC concentrations. Colors are as in **Figure 5B**; otherwise, as in **B**.

(I) Fraction of centrally mismatched target RNAs sliced over time by either wildtype (WT) or central-loop mutants, across different RISC concentrations; otherwise, as in **G**.

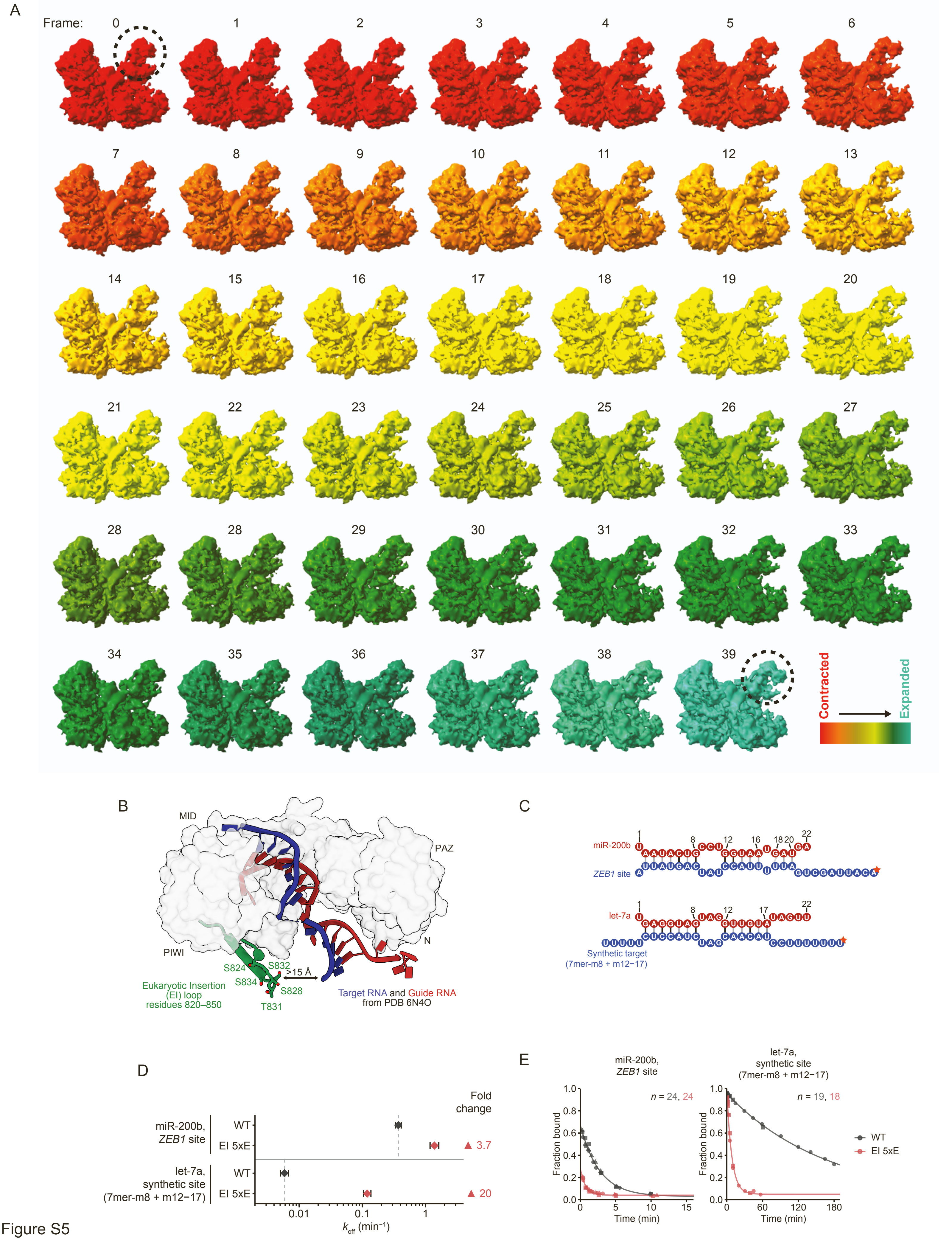
A
Frame:
0
1
2
3
4
5
6
7
8
9
10
11
12
13
14
15
16
17
18
19
20
21
22
23
24
25
26
27
28
28
29
30
31
32
33
34
35
36
37
38
39
Contracted
Expanded
B
MID
PAZ
PIWI
N
S824
S832
S834
>15 Å
S828
T831
Eukaryotic Insertion
(EI) loop
residues 820–850
Target RNA and Guide RNA
from PDB 6N4O
C
miR-200b
ZEB1 site
1
8
12
16
18
20
22
let-7a
Synthetic target
(7mer-m8 + m12−17)
17
D
miR-200b,
ZEB1 site
WT
EI 5xE
let-7a,
synthetic site
(7mer-m8 + m12−17)
Fold
change
▲ 3.7
▲ 20
0.01
0.1
1
k_{off} (min^{-1})
E
miR-200b,
ZEB1 site
let-7a,
synthetic site
(7mer-m8 + m12−17)
n = 24, 24
n = 19, 18
Fraction bound
Time (min)
WT
EI 5xE

Figure S5

Figure S5. Analyses of the central channel expansion and the EI loop, related to Figure 3.
(A) Frames 0−39 of 3DFlex movie (**Movie S4**). Gradient depicts contracted (red) to expanded (cyan) states.
(B) A ~15 Å distance between the EI loop modeled in our HsAGO2 structure and the target RNA from the HsAGO2 two-helix structure (PDB: 6N4O [S1]). The EI loop is shown as a cartoon from residues 820−850, and the rest of HsAGO2 is shown as a surface.
(C) Schematic of miRNA guide–target duplexes examined in target-dissociation assays. The ^{32}P radiolabel at the 5′ end of target is indicated as an orange star. Otherwise, this panel is as in **Figure 1B**.
(D) In vitro dissociation-rate constant (k_{off}) values for either wildtype (WT) HsAGO2 (dark grey) or HsAGO2 with phosphomimetic substitutions in the EI loop (red), for the two miRNA−target sets tested. Otherwise, this panel is as in **Figure 2E**.
(E) Dissociation of target RNA from either wildtype (WT) HsAGO2 or HsAGO2 with phosphomimetic substitutions in the EI loop. Curves represent nonlinear best-fits to an exponential decay equation. Number of data points for each set is indicated as *n*. Time points beyond the limit of the *x* axes are not shown.

Table S1. Oligonucleotides used in this study, related to STAR Methods

**RNA OLIGONUCLEOTIDES**		
**Guide or passenger strands**		
**Sequence**	**Notes**	
/5Phos/rUrGrGrArArGrArCrUrArGrUrGrArUrUrUrUrGrUrUrG	hsa-miR-7	
/5Phos/rArCrArArArArUrCrArCrUrArGrUrCrUrArCrCrArArU	hsa-miR-7*	
/5Phos/rUrGrGrArArGrArCrUrUrGrUrGrArUrUrUrUrGrUrUrG	hsa-miR-7.M1	
/5Phos/rArCrArArArArUrCrArCrArArCrUrCrUrArCrCrArArU	hsa-miR-7.M1*	
/5Phos/rUrArArUrArCrUrGrCrCrUrGrGrUrArArUrGrArUrGrA	hsa-miR-200b	
/5Phos/rArUrCrArUrUrArCrCrArGrGrArArGrUrUrUrUrArArU	hsa-miR-200b*	
/5Phos/rUrGrArGrGrUrArGrUrArGrGrUrUrGrUrArUrArGrUrU	hsa-let-7a	
/5Phos/rCrUrArUrArCrArArUrCrUrArCrUrGrUrCrUrUrUrC	hsa-let-7a*	
/5Phos/rUrGrGrArArGrGrCrUrUrGrUrGrArUrUrGrUrGrUrUrG	hsa-miR-7.M4	
/5Phos/rArCrArCrArArUrCrArCrArArGrUrCrUrArCrCrArArU	hsa-miR-7.M4*	
/5Phos/rUrArGrGrUrArGrUrUrUrCrArUrGrUrUrGrUrUrGrGrG	hsa-miR-196a	
/5Phos/rCrArArCrArArCrArUrGrArArArCrUrArCrUrUrArArU	hsa-miR-196a*	
/5Phos/rUrArGrGrUrArArUrUrUrCrArUrGrUrUrGrUrUrGrGrG	hsa-miR-196a.M2	
/5Phos/rCrArArCrArArCrArUrGrArArUrUrUrArGrCrUrArArU	hsa-miR-196a.M2*	
/5Phos/rUrArArGrUrGrCrUrArUrUrUrGrUrUrGrGrGrGrUrArG	dre-miR-430a	
/5Phos/rArCrCrCrCrArArCrArArArUrUrGrCrArGrUrUrArArU	dre-miR-430a*	
**Target RNAs**		
**Sequence**	**Notes**	
mUmUmUrCrArArCrArArArArUrCrArCrUrArGrUrCrUrUrCrCrAmA mA/35OctdU/	Target RNA in ternary complex for structure characterization	
rArCrArUrUrArGrCrUrGrArUrUrUrUrUrArCrCrUrArUrCrArGrU rArUrUrA	Binding target RNA for hsa-miR-200b	
rUrUrUrUrUrUrUrCrCrUrArCrArArCrGrArUrCrUrArCrCrUrCrU rUrUrUrU	Binding target RNA for hsa-let-7a	
**DNA AND MODIFIED OLIGONUCLEOTIDES**		
**Capture and competitor oligonucleotides for RISC purification**		
**Sequence**	**Notes**	
mAmCmAmUmCmGmUmCmCmGmCmAmCmCmAmCmAmCmGmUmCmUmUmCmCmA mAmCmCmUmUmAmCmAmCmAmC/3Bio/	hsa-miR-7, capture	
AAGGTTGGAAGACGTGTGGTGCGGACGATGT/3Bio/	hsa-miR-7, competitor	
mAmCmAmUmCmGmUmCmCmGmCmAmCmCmAmCmAmCmCmAmGmUmAmUmUmA mAmCmCmUmUmAmCmAmCmAmC/3Bio/	hsa-miR-200b, capture	
AAGGTTAATACTGGTGTGGTGCGGACGATGT/3Bio/	hsa-miR-200b, competitor	
mUmCmUmUmCmCmUmGmCmGmCmAmCmCmAmAmGmCmCmUmAmCmCmUmCmA mAmCmUmUmUmAmCmAmCmAmC/3Bio/	hsa-let-7a, capture	
AAAGTTGAGGTAGGCTTGGTGCGCAGGAAGA/3Bio/	hsa-let-7a, competitor	
mAmCmAmUmCmGmUmCmCmGmCmAmCmCmAmCmAmCmGmCmCmUmUmCmCmA mAmCmCmUmUmAmCmAmCmAmC/3Bio/	hsa-miR-7.M4, capture	
AAGGTTGGAAGGCGTGTGGTGCGGACGATGT/3Bio/	hsa-miR-7.M4, competitor	
mAmCmAmUmCmGmUmCmCmGmCmAmCmCmAmCmAmCmAmCmUmAmCmCmUmA mAmCmCmUmUmAmCmAmCmAmC/3Bio/	hsa-miR-196a, capture	
AAGGTTAGGTAGTGTGTGGTGCGGACGATGT/3Bio/	hsa-miR-196a, competitor	

mAmCmAmUmCmGmUmCmCmGmCmAmCmCmAmCmAmCmAmUmUmAmCmCmUmA mAmCmCmUmUmAmCmAmCmAmC/3Bio/	hsa-miR-196a.M2, capture	
AAGGTTAGGTAATGTGTGGTGCGGACGATGT/3Bio/	hsa-miR-196a.M2, competitor	
mUmCmUmUmCmCmUmCmCmGmCmAmCmCmAmCmAmCmAmGmCmAmCmUmUmA mAmCmCmUmUmAmCmAmCmAmC/3Bio/	dre-miR-430a, capture	
AAGGTTAAGTGCTGTGTGGTGCGGAGGAAGA/3Bio/	dre-miR-430a, competitor	
**IVT templates**		
**Sequence**	**Notes**	**IVT RNA sequence**
gaaatTAATACGACTCACTATAGGG	T7 promoter primer for IVT	
TGTTGTTGTTGTTGTTTGGAAGACTAGTGATTTTGTTGTTGTTGTTGTT GTTGTTGTTGTTGTTCCCTATAGTGAGTCGTATTAATTTC	Perfect slicing target for hsa-miR-7	GGGAACAACAACAAC AACAACAACAACAAC AACAACAAAAUCACU AGUCUUCCAAACAAC AACAACAACA
TGTTGTTGTTGTTGTTTGGAAGACTTGTGATTTTGTTGTTGTTGTTGTT GTTGTTGTTGTTGTTCCCTATAGTGAGTCGTATTAATTTC	mm10AA slicing target for hsa-miR-7, or perfect slicing target for hsa-miR-7.M1	GGGAACAACAACAAC AACAACAACAACAAC AACAACAAAAUCACA AGUCUUCCAAACAAC AACAACAACA
TGTTGTTGTTGTTGTTTGGAAGACTACTGATTTTGTTGTTGTTGTTGTT GTTGTTGTTGTTGTTCCCTATAGTGAGTCGTATTAATTTC	mm11GG slicing target for hsa-miR-7	GGGAACAACAACAAC AACAACAACAACAAC AACAACAAAAUCAGU AGUCUUCCAAACAAC AACAACAACA
TGTTGTTGTTGTTGTTTGGAAGGCTTGTGATTGTGTTGTTGTTGTTGTT GTTGTTGTTGTTGTTCCCTATAGTGAGTCGTATTAATTTC	Perfect slicing target for hsa-miR-7.M4	GGGAACAACAACAAC AACAACAACAACAAC AACAACACAAUCACA AGCCUUCCAAACAAC AACAACAACA
TGTTGTTGTTGTTGTTTAGGTAGTTTCATGTTGTTGGGTTGTTGTTGTT GTTGTTGTTGTTGTTCCCTATAGTGAGTCGTATTAATTTC	Perfect slicing target for hsa-miR-196a	GGGAACAACAACAAC AACAACAACAACAAC AACCCAACAACAUGA AACUACCUAAACAAC AACAACAACA
TGTTGTTGTTGTTGTTTAGGTAATTTCATGTTGTTGGGTTGTTGTTGTT GTTGTTGTTGTTGTTCCCTATAGTGAGTCGTATTAATTTC	Perfect slicing target for hsa-miR-196a.M2	GGGAACAACAACAAC AACAACAACAACAAC AACCCAACAACAUGA AAUUACCUAAACAAC AACAACAACA
TGTTGTTGTTGTTGTTTAAGTGCTATTTGTTGGGGTAGTTGTTGTTGTT GTTGTTGTTGTTGTTCCCTATAGTGAGTCGTATTAATTTC	Perfect slicing target for dre-miR-430a	GGGAACAACAACAAC AACAACAACAACAAC AACUACCCCAACAAA UAGCACUUAAACAAC AACAACAACA
**Plasmid cloning**		
**Sequence**	**Notes**	
AGAGGATCCTACTCGGGAGCCGG	SUMO_Eu1 insertion, backbone PCR, F, BamHI	
AGAGCTAGCACCACCAAGCTTGTCATCG	SUMO_Eu1 insertion, backbone PCR, R, NheI	
AGAGCTAGCggatctgctgcgggtg	SUMO_Eu1 insertion, insert PCR, F, NheI	
AGAGGATCCgcctccagtctggtgcag	SUMO_Eu1 insertion, insert PCR, R, BamHI	
cgcatcatcttctaccgcgAcggtgtctctgaaggccag	HsAGO2 site-directed mutagenesis: restore D669, F	
ctggccttcagagacaccgTcgcggtagaagatgatgcg	HsAGO2 site-directed mutagenesis: restore D669, R	
GGACATCCCCAAAATTGACATCTATgcTTATGAATTGGATATCAAGCCAGAG	HsAGO2 site-directed mutagenesis: H56A, F	
CTCTGGCTTGATATCCAATTCATAAgcATAGATGTCAATTTTGGGGATGTCC	HsAGO2 site-directed mutagenesis: H56A, R	
CAAGCCAGAGAAGTGCCCGgccAGAGTTAACAGGGAAATCGTGG	HsAGO2 site-directed mutagenesis: R68A, F	
CCACGATTTCCCTGTTAACTCTggcCGGGCACTTCTCTGGCTTG	HsAGO2 site-directed mutagenesis: R68A, R	
GAAGCCCGTGTTTGACGGCgcGAAGAATCTATACACAGCCATGC	HsAGO2 site-directed mutagenesis: R97A, F	
GCATGGCTGTGTATAGATTCTTCgcGCCGTCAAACACGGGCTTC	HsAGO2 site-directed mutagenesis: R97A, R	

GAAGCCCGTGTTTGACGGCAGGgcgAATCTATACACAGCCATGCCC	HsAGO2 site-directed mutagenesis: K98A, F	
GGGCATGGCTGTGTATAGATTcgcCCTGCCGTCAAACACGGGCTTC	HsAGO2 site-directed mutagenesis: K98A, R	
GGACATCCCCAAAATTGACATCTATGCTTATGAATTGGATATCAAGCCAGAG AAGTGCCCGGCCAGAGTTAACAGGGAAATCGTGG	HsAGO2 site-directed mutagenesis: H56A+R68A, F	
CCACGATTTCCCTGTTAACTCTGGCCGGGCACTTCTCTGGCTTGATATCCAA TTCATAAGCATAGATGTCAATTTTGGGGATGTCC	HsAGO2 site-directed mutagenesis: H56A+R68A, R	
GAAGCCCGTGTTTGACGGCGCGGCGAATCTATACACAGCCATGCCC	HsAGO2 site-directed mutagenesis: R97A+K98A, F	
GGGCATGGCTGTGTATAGATTCGCCGCGCCGTCAAACACGGGCTTC	HsAGO2 site-directed mutagenesis: R97A+K98A, R	
GAAGCCCGTGTTTGACGGCGAGGAGAATCTATACACAGCCATGCCC	HsAGO2 site-directed mutagenesis: R97E+K98E, F	
GGGCATGGCTGTGTATAGATTCTCCTCGCCGTCAAACACGGGCTTC	HsAGO2 site-directed mutagenesis: R97E+K98E, R	
CACCTTCATCGTGGTGCAGAAGgcGCACCACACCCGGCTCTTCTG	HsAGO2 site-directed mutagenesis: R710A, F	
CAGAAGAGCCGGGTGTGGTGCgcCTTCTGCACCACGATGAAGGTG	HsAGO2 site-directed mutagenesis: R710A, R	
CACCTTCATCGTGGTGCAGAAGAGGCACGCCACCCGGCTCTTCTGCACTG	HsAGO2 site-directed mutagenesis: H712A, F	
CAGTGCAGAAGAGCCGGGTGGCGTGCCTCTTCTGCACCACGATGAAGGTG	HsAGO2 site-directed mutagenesis: H712A, R	
CACCTTCATCGTGGTGCAGAAGgcGCACgcCACCCGGCTCTTCTGCACTG	HsAGO2 site-directed mutagenesis: R710A+H712A, F	
CAGTGCAGAAGAGCCGGGTGgcGTGCgcCTTCTGCACCACGATGAAGGTG	HsAGO2 site-directed mutagenesis: R710A+H712A, R	
GCGCGTGCAGCAGCACGCCCAGGAGATCATACAAGACCTGGC	HsAGO2 site-directed mutagenesis: R635A, F	
GCCAGGTCTTGTATGATCTCCTGGGCGTGCTGCTGCACGCGC	HsAGO2 site-directed mutagenesis: R635A, R	
CCAGGTACCACCTGGTGGATAAGGAACATGACGAGGCTGAAGGAGAGCATGA GGAGGGGCAGGAGAACGGGCGAGACCACCAAG	HsAGO2 site-directed mutagenesis: EI 5xE, F	
CTTGGTGGTCTCGCCCGTTCTCCTGCCCCTCCTCATGCTCTCCTTCAGCCTC GTCATGTTCCTTATCCACCAGGTGGTACCTGG	HsAGO2 site-directed mutagenesis: EI 5xE, R	

Table S2. Modeling of complex, related to Figures 1–5.

Domain / Chain ID	Domain/ region	Residue range	Initial Model	PDB template for initial model/ chain	Map used for modeling	Modeling algorithm	Changes to initial model	Confidence of modeling	Relative map resolution
**N-term/A**	N-term	23–52	Crystal Structure	4OLA	2	Rigid body fitting	Manual correction, phenix.real_space_refine	Atomic model	3–8 Å
**N/A**	N	53–140	Crystal Structure	4OLA	2	Rigid body fitting	Manual correction, phenix.real_space_refine	Atomic model	6–8 Å
**L1/A**	L1	141–229	Crystal Structure	4OLA	2	Rigid body fitting	Manual correction, phenix.real_space_refine	Atomic model	6–8 Å
**PAZ/A**	PAZ	230–348	Crystal Structure	4OLA	2	Rigid body fitting	Manual correction, phenix.real_space_refine	Atomic model	6–8 Å
**L2/A**	L2	349–444	Crystal Structure	4OLA	2	Rigid body fitting	Manual correction, phenix.real_space_refine	Atomic model	3–8 Å
**MID/A**	MID	445–577	Crystal Structure	4OLA	2	Rigid body fitting	Manual correction, phenix.real_space_refine	Atomic model	3–8 Å
**PIWI/A**	PIWI	578–821, 845–859	Crystal Structure	4OLA	2	Rigid body fitting	Manual correction, phenix.real_space_refine	Atomic model	3–8 Å
**PIWI/A**	PIWI/Eukaryotic Insertion Loop	822–844	Alphafold2	N/A	2	Alphafold2	Manual correction, phenix.real_space_refine	Alphafold2	6–8 Å
**Guide RNA/G**	Guide RNA	1–8	Crystal Structure	6N4O	2	Rigid body fitting	Manual correction, phenix.real_space_refine	Atomic model	3–8 Å
**Guide RNA/G**	Guide RNA	9–21	ChimeraX generated	N/A	2	Rigid body fitting	Manual correction, phenix.real_space_refine	Pseudo-atomic model	3–8 Å
**Target RNA/T**	Target RNA	15–22	Crystal Structure	6N4O	2	Rigid body fitting	Manual correction, phenix.real_space_refine	Atomic model	3–8 Å
**Target RNA/T**	Target RNA	1–14	ChimeraX generated	N/A	2	Rigid body fitting	Manual correction, phenix.real_space_refine	Pseudo-atomic model	3–8 Å

Supplemental references

1. Sheu-Gruttadauria, J., Xiao, Y., Gebert, L.F., and MacRae, I.J. (2019). Beyond the seed: structural basis for supplementary microRNA targeting by human Argonaute2. EMBO J., e101153. https://doi.org/10.15252/embj.2018101153.
2. Sheng, G., Zhao, H., Wang, J., Rao, Y., Tian, W., Swarts, D.C., Oost, J. van der, Patel, D.J., and Wang, Y. (2014). Structure-based cleavage mechanism of Thermus thermophilus Argonaute DNA guide strand-mediated DNA target cleavage. Proc. Natl. Acad. Sci. *111*, 652–657. https://doi.org/10.1073/pnas.1321032111.
3. Xiao, Y., Maeda, S., Otomo, T., and MacRae, I.J. (2023). Structural basis for RNA slicing by a plant Argonaute. Nat. Struct. Mol. Biol., doi:10.1038/s41594-023-00989-7. https://doi.org/10.1038/s41594-023-00989-7.
4. Doxzen, K.W., and Doudna, J.A. (2017). DNA recognition by an RNA-guided bacterial Argonaute. PLOS ONE *12*, e0177097. https://doi.org/10.1371/journal.pone.0177097.
5. Miyoshi, T., Ito, K., Murakami, R., and Uchiumi, T. (2016). Structural basis for the recognition of guide RNA and target DNA heteroduplex by Argonaute. Nat. Commun. *7*, 11846. https://doi.org/10.1038/ncomms11846.
6. Schirle, N.T., Sheu-Gruttadauria, J., Chandradoss, S.D., Joo, C., and MacRae, I.J. (2015). Water-mediated recognition of t1-adenosine anchors Argonaute2 to microRNA targets. eLife *4*. https://doi.org/10.7554/eLife.07646.
7. Wang, Y., Juranek, S., Li, H., Sheng, G., Wardle, G.S., Tuschl, T., and Patel, D.J. (2009). Nucleation, propagation and cleavage of target RNAs in Ago silencing complexes. Nature *461*, 754–761. https://doi.org/10.1038/nature08434.
